# Automation in the pharmaceutical analysis laboratory: a centralized/decentralized approach

**DOI:** 10.1155/S1463924695000071

**Published:** 1995

**Authors:** Stephen Scypinski, Linda Nelson, Theodore Sadlowski

**Affiliations:** Pharmaceutical Analysis Research and Development Hoffmann-La Roche Inc. Nutley NJ 07110 USA

## Abstract

It has been over 10 years since robots have appeared in the pharmaceutical analysis laboratory. In the early days, it was common for one selected individual to be responsible for the programming, usage and maintenance of the robots(s). However, the increasing use of robotics has prompted the formation of robotics ‘laboratories’ and/or ‘groups’. This is especially true when multiple robotic systems and applications are involved.

Over the past several years at ISLAR, many champions of robotics have given presentations on the setup and usage of robotics within their organizations. These managers have described both the ‘centralized’ and ‘decentralized’ approaches to the implementation of robotics. In the centralized system, a single group is charged with all aspects of the robotic project, including justification, purchase, validation, use and maintenance. Under such an arrangement, samples are usually given to the robotics group for analysis. In contrast, a totally decentralized approach to robotics would have units interspersed throughout the organization, with each individual group responsible for their respective unit(s), in much the same way as liquid chromatographs are considered.

At Hoffmann-La Roche, aspects of both the centralized and decentralized approaches to robotics are used which make our combined system the ‘best of both worlds’. This paper describes the Roche philosophy towards robotics and highlights the advantages to the system used.


Journal of Automatic Chemistry, Vol. 17, No. 2 (March–April 1995), pp. 47–49

### Automation in the pharmaceutical analysis laboratory: a centralized/decentralized approach

**Stephen Scypinski, Linda Nelson and Theodore Sadlowski**

*Pharmaceutical Analysis Research and Development, Hoffmann-La Roche Inc., Nutley, NJ 07110, USA*

*It has been over 10 years since robots have appeared in the pharmaceutical analysis laboratory. In the early days, it was common for one selected individual to be responsible for the programming, usage and maintenance of the robots(s). However, the increasing use of robotics has prompted the formation of robotics 'laboratories' and/or 'groups'. This is especially true when multiple robotic systems and applications are involved.*

*Over the past several years at ISLAR, many champions of robotics have given presentations on the setup and usage of robotics within their organizations. These managers have described both the 'centralized' and 'decentralized' approaches to the implementation of robotics. In the centralized system, a single group is charged with all aspects of the robotic project, including justification, purchase, validation, use and maintenance. Under such an arrangement, samples are usually given to the robotics group for analysis. In contrast, a totally decentralized approach to robotics would have units interspersed throughout the organization, with each individual group responsible for their respective unit(s), in much the same way as liquid chromatographs are considered.*

*At Hoffmann-La Roche, aspects of both the centralized and decentralized approaches to robotics are used which make our combined system the 'best of both worlds'. This paper describes the Roche philosophy towards robotics and highlights the advantages to the system used.*

### Introduction

The use of automation in the pharmaceutical analysis laboratory is reaching a level of maturity. The regular attendees at ISLAR have heard many and varied stories on the implementation of robotics within analytical laboratories: presentations have addressed equipment justification, time savings and impact on analytical personnel. In the session on 'managing laboratory automation' laboratory managers learnt the proper strategy for such key aspects of automation as the selection of personnel and use of the systems can be the difference between success and failure of the automation project.

Part of the challenge is the high visibility of the laboratory robot. Although one can argue that cost is an issue, many other laboratory instruments, most notably nuclear magnetic resonance and mass spectrometers, cost much more than even a fully configured PyRobotics system, yet it is highly unlikely that the upper level management that approved the requisition for that purchase will ever question its utility or time saving potential. However, as most of us know, the person who justifies and convinces management within his or her organization to purchase a robotics system can be sure to receive frequent reminders from their superiors that this purchase promised to improve sample throughput or reduce sample backlog. For this reason, the proper system for insuring that the robot is not only used, but receives the attention it needs to show the greatest return on investment, must be implemented. The system for operating a robot or, in the case of several robots, a robotics 'group' or 'laboratory' provides the interface between upper level management and the real work being performed by the robot. It is critically important that the person or persons in this group be of high calibre and realize the important service to the reputation of the laboratory that they fulfill.

In my several years of involvement with laboratory automation, I have seen many colleagues actually fail, or at the very least succeed only to the point of that they became disenchanted with robotics. In most cases this was due to extreme caution practiced by those individuals who convinced their management of their companies to take the plunge and purchase their first robotic system. As most scientists are inherently cautious (a drawback from all the years of being taught to approach problems in a logical fashion), they took it on themselves to justify the purchase and convince management of its validity. When the system arrived, they felt that it was their 'baby' and took it upon themselves to singlehandedly set up, program and utilize the new robot. The problem arises as an individual such as this normally has other obligations with respect to their job. For example, even a Ph.D. chemist in a non-supervisory capacity is expected to attend meetings and make presentations about the projects to which they are currently assigned. Without the ability to devote full time to the robot, projects usually linger, or reach the 'back burner' status. In many instances, the robot becomes an unused relic kept in some back room.

The past history of robotic implementation in analytical development laboratories has not been all grim. There have been many great success stories of individuals who managed not only to implement a single robot, but have also shown the ability to grow and expand the usage of automation in the analytical laboratory. The automation 'specialist' was the person who most often contributed to the successful transfer of a manual analytical method to a robotic system. The automation specialist was that unique individual who not only had the correct blend of analytical background, computer literacy and ability to work with instrumentation, but also had the time to contribute to taking the project to fruition. Indeed, as

This paper was presented at the 1994 ISLAR meeting.

© Zymark Corporation 1995

specialist would be unique for a time, but would eventually become commonplace. At ISLAR 1984, Frank Zenie said that: 'Automation specialists are emerging as a new function in the laboratory. This evolution is similar to that which created the need for chemical engineers and, more recently, computer specialists. In the future, as this technology becomes widely used, these specialists will disperse back into the operating organization' [1]. This was proven to be true. In a plenary address at ISLAR 1992, Dr Eugene McGonigle of Schering-Plough pointed out that the evolution of robotics within his organization has resulted in automated methods being developed by 'all laboratory staff, not a specially trained few' [2]. This implies that the natural progression of a new technology, such as robotics, into an existing analytical organization which is keyed in to new technology has the new technique first utilized by a select few individuals, followed by everyone in the laboratory. Indeed, there are many who liken the development of robotics to that of high performance liquid chromatography (HPLC) in the late 1960s and early 1970s.

When faced with the task of successfully implementing robotics within the Pharmaceutical Analysis Laboratories of Hoffmann-La Roche, we sought to use the evidence of the past to guide us in forming the proper strategy for the utilization of this technique.

### Robotics at Hoffman-La Roche

The Pharmaceutical Analysis Research and Development (PARD) section of the Pharmaceutical Quality Control and Quality Assurance Department serves as the total analytical support function for the development of drug substance synthetic processes and the corresponding dosage forms. The primary responsibilities of PARD are:

(1) Develop/validate analytical methods and test procedures for drug substances, dosage forms (formulations), packaging components, intermediates, blends, granulations etc.

(2) Provide release and stability testing (support) for clinical materials. Serve as the 'quality unit' for research compounds and products.

(3) Perform analytical investigations in conjunction with other departments.

(4) Prepare regulatory documentation (INDs/NDAs/amendments/supplements).

(5) Perform analytical research as it relates to new analytical technology/techniques/methods for pharmaceutical analysis. Publish/present results of experiments/studies.

(6) Participate in regulatory inspections and answer FDA questions.

(7) Support various development departments within the R&D framework of the company.

In the Roche project team approach to development, analytical R&D is considered a true partner in drug development. For this reason, individuals from PARD serve on international project teams which are charged with moving a project along its development pathway. The challenge within the analytical R&D setting is to implement systems and techniques to help meet the aggressive deadlines imposed by project management. Laboratory automation is one way in which the operation has been streamlined. While there are many reasons to automate the major reasons for moving in this direction are:

(*a*) Superior productivity.

(*b*) Ability to perform more complex test procedures.

(*c*) 'Just in time' analyses.

(*d*) Improved precision of analysis.

(*e*) Reduce cost of analysis.

(*f*) Efficient transfer to multiple sites.

(*g*) Effective use of space.

(*h*) Documentation.

(*i*) Safeguard personnel from highly potent and somewhat unknown substances.

(*j*) Ability to analyse highly unstable and photolabile materials.

In trying to achieve these results, we tried to ascertain which manner of laboratory or group would best serve our needs. The choices were limited to: centralization of the robotics function; total decentralization; some combination of the first two.

In a centralized laboratory, all robotic systems are under the control of a single person or group. A common example of a centralized function in most analytical laboratory setups is nuclear magnetic resonance spectroscopy (NMR). Most analysts working in the development of new products or processes do not run their own NMR spectra, but rather submit samples to the NMR laboratory, who provides the services of running the sample, providing a spectrum and corresponding interpretation. The advantages of a centralized laboratory are that all equipment is operated in the same fashion. In addition, it is validated and maintained at the same level of compliance. The major disadvantage of the application of this approach to managing a robotics laboratory is that the continuing success of the operation depend on a small group of individuals who hold the 'keys to the kingdom'. There have been several situations where the lead person (automation specialist) was lured away by a competing organization. This can be disastrous to an automation effort as it is difficult to keep such a program underway without a key player to drive the project.

Under a decentralized structure, all personnel working in a laboratory have access to or perform a specific technique on a routine basis. The most common example of a decentralized technique is high performance liquid chromatography (HPLC). While this overcomes the disadvantage of having only a select individual possess all the knowledge needed to pursue robotics, the drawback is that all users are free to exercise their own judgement with respect to implementing automated methods. In addition, one true source of training and guidance is not available under a decentralized system.

The hybrid approach at Hoffmann-La Roche combines the best characteristics of both the centralized and decentralized systems of robotics. The specific positive aspects of the combined approach are:

(1) Equipment is maintained and validated in a uniform manner by a central group. Standard Operating Procedures (SOPs) are written and enforced by this group.

(2) Robotic systems are accessible and usable by other members of the laboratory for both development work as well as routine analysis.

(3) Automation projects have a high probability of success under this system, as a mix of robotics expertise and specific chemistry background contribute to and reinforce the project.

(4) The people working on the project benefit from the sharing of ideas and experience.

(5) The individuals and/or groups can obtain ownership of a project without actually having to directly supervise individuals.

Under the combination approach, assigned individuals from both the robotics group and the specific analytical area work together to develop and validate automated methods for developmental products. It is also the combined efforts of both individuals which produce the required documentation, including the pertinent portions of the regulatory submission, necessary to show that the automated method is indeed successful.

The robotics laboratory at Hoffman-La Roche is also charged with the development and validation of methods for marketed products as well. In this age of 'less with more', we identified several high-volume marketed products which would benefit from their application of robotics, either because the method is simple and the sheer volume of samples makes the use of automation attractive, or the analysis is complex and requires special considerations, such as the stipulation that the analysis must be carried out in the dark due to the photolability of the sample. There are several products which fit either of the two catagories and these manual methods are being converted to automated procedures. It is hoped to create 'turnkey analysers', which may be easily used by all laboratory personnel with some experience in basic analytical chemistry. It should be pointed out here that another ploy to ensuring the success of robotics within an organization is to make it accessible, i.e. usable, by as large a population as possible.

A key to the success of the implementation of robotics in an analytical development laboratory is the 'building in' of the capability of converting to robotics even in the manual methods. For example, when working up a sample preparation procedure for a tablet assay method, it is important to add several intact tablets to a specified volume of diluent followed by some sort of agitation. This mixture is then filtered and diluted prior to analysis by HPLC. This procedure is easily transferred to the robot without the need to perform lengthy equivalency studies between incompatible procedures, such as the use of grinding several tablets together and adding a single tablet weight to the diluent prior to HPLC injection. One of the charges of the robotics personnel is to impress upon the method development chemists the need to consider the use of automation, even though it may be several years before the sample volume makes the use of robotics attractive.

The key to the success of such a program as has been described here is the personnel. The proper candidate for the robotics laboratory has the following qualifications and background:

(1) A solid foundation in analytical chemistry, including method development and validation. The individual should preferably have come from the development laboratory.

(2) Computer literate and willing to learn about new areas.

(3) Not be adverse to getting dirty hands and working with mechanical things.

(4) Have good interpersonal skills.

(5) Not be connected with other duties, especially those of an administrative nature.

(6) Finally, they must be very patient.

### Conclusions

The centralized/decentralized concept of robotics has been in place at Hoffmann-La Roche for approximately three years. It is working well within the organization and has been applied to the functionalization of other areas, such as gas chromatography.

### References

1. Zenie, F. H., In *Proceedings of the 1984 International Symposium on Laboratory Automation and Robotics* (1984), 1, pp. 1–16.
2. McGonigle, E. J., In *Proceedings of the 1992 International Symposium on Laboratory Automation and Robotics* (1992), 9, pp. 1–15.

